# FEMINIST TO POSTFEMINIST: *contemporary biofictions by and about women artists*

**DOI:** 10.1080/0969725X.2017.1286090

**Published:** 2017-03-17

**Authors:** Julia Novak

**Affiliations:** ^a^ Department of English and American Studies, University of Salzburg, Erzabt-Klotz-Straße 1, 5020Salzburg, Austria

**Keywords:** biofiction, biographical novel, historical romance novel, feminist biography, metabiography, Priya Parmar, Janice Galloway

## Abstract

Biographical novels about historical women artists have been experiencing a veritable boom in recent years. Written mostly by women, they can be understood as women authors’ attempts to reach out across time (and often, space) to other “artistic” women whose lives “speak to us” today. It has long been a key insight of historical fiction research that a historical novel reveals more about the time in which it was written than the time in which it is set. As such, it can be assumed that contemporary novels about historical women speak as much to twenty-first-century conceptions of femininity as to particular historical moments of female subjectivity. This paper will compare two novels about historical women artists: Janice Galloway’s *Clara* (2002) about nineteenth-century German pianist Clara Wieck-Schumann and Priya Parmar’s *Exit the Actress* (2011) about Restoration actress Nell Gwyn. While based on historical facts, both these novels use the greater freedom of fiction to depart from biographical conventions. It will be demonstrated that although they resemble each other on the discourse level, employing shifts in the narrative perspective, conspicuous typography, and graphic elements, they differ markedly in the biographical and fictional subgenres in which they participate and, hence, in their gender politics.

Biographical novels about women artists have been experiencing a veritable boom in recent years. Since the turn of the millennium, the lives of Frida Kahlo, Sylvia Plath, Murasaki Shikibu, Artemisia Gentileschi, Marilyn Monroe, Jane Austen, Gwen John – to name just a few – have been made subjects of imaginative re-tellings, some of them in multiple versions. These biofictions are the product of both biographical scholarship and authorial imagination. As most of them are written by women, they also mark women authors’ attempts to reach out across time (and often, space) to other “artistic” women whose lives “speak to us” today. Thus, while for Martin Middeke (3) self-reflexivity constitutes a chief characteristic of postmodern biofictions in general, novels by and about women artists are self-reflexive in two ways: as narratives of artistry and femininity. This paper compares two novels – Janice Galloway’s *Clara* (2002) about nineteenth-century German pianist Clara Wieck-Schumann and Priya Parmar’s *Exit the Actress* (2011) about Restoration actress Nell Gwyn – in view of the particular images of women artists they present.

While contemporary biofictions about historical women artists must be situated in the context of a general boom in biofiction since the 1980s,^1^ the origins of this trend can also be traced to the mid-twentieth-century surge in biographies about “notable” women of the past, as part of the so-called second wave of feminism. Reacting against Thomas Carlyle’s famous dictum that “the history of the world is but the biography of great men” (Carlyle 26), feminist biographers aimed to “restore” women to the historical record. Many biographers aimed specifically at making women’s achievements visible, and this strand of exemplary feminist biography still continues today – in individual women’s biographies as well as in inspirational collective biographies such as Caroline Criado-Perez’s recent *Do It Like a Woman … and Change the World*. Their purpose is to offer readers stories about exceptional women “reinventing what it means to be female” (“Do It Like a Woman” n. pag.).

Like these exemplary feminist biographies, the overwhelming majority of women artist biofictions centres on canonical artists and, hence, on famous, “exceptional” women, whose lives can teach us something about “reinventing what it means to be female.” And as in biography, the image of the woman artist emerging from a novel depends on the particular life narrative the writer constructs on the basis of the available material. It has long been a key insight of historical fiction research that a historical novel reveals more about the time in which it was written than the time in which it is set. As such, it can be assumed that contemporary biofictions about women speak as much to twenty-first-century conceptions of femininity as to particular historical moments of female subjectivity. And again, similar arguments have been put forward about “factual” biography.^2^

However, unlike the biographer, the novelist is not bound by historical fact and biographical convention. As products of the postmodern disregard for generic boundaries, biofictions can thus participate in biographical as well as in fictional subgenres and align their structure, narrative mode and character properties with the requirements of these subgenres. Given the function of genre as a power discourse that contributes to the “social structuring of meaning” (Frow 1), female subjectivities in biofictions can be related to the specific subgenres that come into play in the texts. In the following, Janice Galloway’s novel *Clara* and Priya Parmar’s *Exit the Actress* will be examined with regard to the relation between their generic layering and their gender politics. Both novels are conspicuous for their formal experiments: they employ shifts in the narrative perspective, unusual typography, and graphic elements. The primary impulse for this paper came from my curiosity to find out how these formal experiments relate to the two novels’ generic make-up – to the genres in which they participate – and to particular visions of femininity which these entail.

## re-writing clara wieck-schumann

Janice Galloway’s fictional account of the life of German pianist Clara Wieck-Schumann covers the artist’s early childhood and the musical training she received from her father Friedrich Wieck, the legendary legal battle between Friedrich Wieck and composer Robert Schumann who was to be her husband, the years of the Schumanns’ marriage, and ends on Robert Schumann’s death in 1856.

When Clara is eighteen, she has her international breakthrough in Vienna. The following quotation illustrates the tone of Galloway’s narrative:

Poor Herr Wieck! He bawls like a baby and blasts his capacious nose into his handkerchief, has to sit for support. A foreigner, a Protestant, a *girl* – he had thought every fibre of Austrian tradition worked against it; yet there she is, Royal and Imperial Chamber Virtuosa to the Austrian Court, glittering with medals, and his own, entirely, wholly, legally and morally his own. (Galloway 154–55)

Friedrich Wieck’s claim to ownership over his daughter is emphasized and clearly ironized in the novel, which functions as a feminist comment. Galloway’s approach to her heroine is generally marked by a focus on gender norms, which is a chief characteristic of feminist biography. “When the subject is female, gender moves to the center of the analysis” (7), Sara Alpern notes in her introduction to *The Challenge of Feminist Biography*. And, indeed, Galloway’s Clara Wieck is continuously positioned within the sex/gender-system of her day by the narrator; her actions are explicitly assessed with regard to prevailing gender norms, which are then implicitly or explicitly critiqued.

Clara emerges as a talented and ambitious artist who enjoys performing, who initially finds herself controlled by her father, for whom her talent has become a major source of income, and then constrained by her overbearing, mentally unstable husband: “Not that he will stop her playing,” he notes, “not at all! She will play endlessly, he hopes, but suitably, appropriately, at home” (Galloway 156). The following scene occurs after one of the Schumanns’ house parties:

He returned some hours later when she was already in bed, and had sex with her as immoderately and repeatedly as he had earlier praised her. Do not play so much, he said abruptly when it was done. The soul sickens of too much. If it had not been for my eyes willing it to stop, you should have driven me from the house. (200)

Consequently, Galloway’s heroine enacts what has been identified as a classic trope of feminist biography: the overcoming of obstacles in her path to self-actualization and artistic independence (Ní Dhúill 116). At one point the narrator asks:

And was it worth it? All the anguish and isolation of selling her playing abroad, of leaving her spouse and child, of braving storms and ships and the ghastly business of organisation all alone? Was it? No one would have asked Liszt such a question. Or Thalberg or Henselt or Mendelssohn. No matter. No one passed up the opportunity to ask Clara Schumann. (Galloway 236)

This passage serves, on the one hand, to drive home to the reader the cultural significance of Clara Wieck-Schumann’s achievement – her name is up there with all the musical greats of her day – and simultaneously, to highlight the different standards to which women were held in terms of their domestic responsibilities.^3^ Some of the numerous lists that constitute one of the novel’s conspicuous graphic features perform a similar function. Clara returns from a concert tour which she has undertaken without her husband:

hers The chopping of cabbage. First teeth.

  Hire of new housemaid, interviews, checking of references.

  Listing of all household stock.

  Local concert for Hamburg fire victims.

  Accounts. And silence. Much silence.

his  A minor. F major. A major.

  Three quartets in as many weeks.

  Three children barely born, yet beautiful.

She was home.

(Galloway 237)

However, a feminist biography not only addresses “woman’s condition in history” (Gutiérrez 54) – as Galloway’s narrative clearly does – but, according to Liz Stanley, it also adopts a metabiographical stance: it “textually recognizes that its facts and arguments are contingent” and that “biographical as any other writing is produced from a particular viewpoint” (250). Galloway’s *Clara* contains an interesting example – a letter – in which the typography enacts a self-reflexive gesture towards biography’s limitations:

**Dear Mother**

**You have as yet heard Nothing from me but now I can write a little** a little is it modest to say a little **I will send you** Herr Bargiel is it impolite not to mention Herr **a Letter which will please you** will be proud of my spelling I am trying my handwriting

[…]

Scales a ruler under my wrist passing the thumb beneath **I was not at all frightened but the Clapping troubled me** my bow is not good I am ungainly and embarrassed to bow so close in our own parlour **Emilie Reichold and M Kupfer played too** Emilie looks like an angel she is blonde where I am **The day before my Birthday I went to Malger with father** father is reading this **Please give my Love to Grandmama and my Brothers.** (Galloway 62–63)

The words in bold are what the child Clara actually writes down. As in other instances where Galloway includes lines from letters, the way these are set off from the rest of the text – italicized or indented – points to the novel’s intertextuality, to the fact that it is based on, and quotes from, historical documents. In this sense, typography communicates authenticity: it stages the text’s reference to real-world characters and events.^4^ Besides lists and letters, *Clara* also contains samples of musical notation and concert programmes that are visually set off from the text and have a similar function: as markers of authenticity they evoke the historical artist’s experiences and achievements.

In the example of the above letter, however, the small print in between the larger words imagines the thoughts and constraints Clara experiences in the act of writing (“father is reading this”). Clara’s inability to translate her feelings and experience into words is, in fact, a recurrent motif in Galloway’s novel. This letter equally points to the selective and refracted nature of what *is* written down as to what is omitted. Its typographical variation thus visualizes the unreliability of the available records on which any biographical endeavour must depend. The novel can be conceived as a “fictional metabiography,” to use Ansgar Nünning’s term (202): it draws attention to the epistemological challenges of life-writing and, ultimately, questions the possibility of distinguishing fact from fiction. In this example, then, the postmodernist variant of the biographical novel converges with feminist biography, as Clara’s deliberate or imposed silences reflect a central feminist concern: the inaccessibility of female experience as a subject of biography (Ní Dhúill 113).

The novel already hints at its metabiographical framework in the fictive concert programme at its very beginning, which announces a performance of Robert Schumann’s opus 42 *Frauenliebe und -Leben* – Woman’s Life and Love – as a “*revised edition of eight songs with piano accompaniment*,” and whose end reads:

*Finale*

Concerto for lone piano

Work in progress

(Galloway 3)

The qualifier “revised” can be read in two ways: it can point to the fact that Clara Wieck-Schumann’s life as such departed markedly from the story Schumann’s song cycle tells about “woman’s life”; more likely, though, it can be read as a programmatic statement on Galloway’s own narrative about that life, which places Clara centre stage. While Galloway’s creative paratext thus points to the ways in which representations of the pianist have traditionally been circumscribed by her husband’s work, it ends on a “concerto for lone piano” and “work in progress” (3), also suggesting a provisional quality, a fundamental unfinishedness, of biographies about women.

## nell gwyn: mad about the king

Priya Parmar’s *Exit the Actress* opens with an adolescent Nell Gwyn who is beginning to write a diary in 1662. It follows the years in which she is discovered by the King’s Company at Theatre Royal, Drury Lane, where she becomes one of the very early star actresses on the Restoration stage, and closes on her retreat from the stage once she has established herself as mistress to King Charles II.

The visual appearance of Parmar’s text is similarly fragmented as Galloway’s. Nell’s diary entries alternate with articles from the *London Gazette*, “London’s Best and Brilliant Broadsheet” (16), echoing the tone of contemporary gossip columns, with notes from the Secretary of State, Lord Arlington (underlain in grey), theatre programmes, and letters to and from other characters, such as Queen Henrietta Maria, King Charles II, and his sister Minette, which have ornamental frames drawn around them. As in Galloway’s novel, Parmar’s inclusion and visual separation of life-writing formats such as letters and diary entries gesture towards the factual basis of her narrative. However, while the visual fragmentation of Galloway’s *Clara* coincides with its deconstruction of traditional gender norms, *Exit the Actress* cannot as easily be subsumed under the label of feminist biography, as I will demonstrate.

Like Galloway’s *Clara*, Parmar’s novel opens with a metafictional gesture: it presents a playbill listing the narrative’s “cast,” and, subsequently, a “Prologue spoken by the actress Mrs. Nelly Gwyn upon her Farewell Performance” (5–7), which closes as follows:

Young girls ask how did you do it? Your cheeks are so pink? Your hair is so *red*? True, you are a stage delight, your waist is slim, your tread is light – but is that *all*? After all, you are so *small*. You are so like us. So here. So wicked. And yet, he loves you so. Why?

(*Quietly.*) And the answer is always the same: I really do not know. (7)

“How did you do it?” How did she manage to capture the heart of the king? This passage anticipates what can be identified – despite its narrative fragmentation – as the novel’s overall trajectory: the heroine’s successful quest for true love. Superficially, *Exit the Actress* could therefore be aligned with the historical romance novel: its beautiful, spirited young heroine enacts the Cinderella-like ascent typical of the genre, winning the love and devotion of an aristocratic, saturnine hero (as well as his economic support) – in a period that is historically removed and therefore suitably “romantic” as a setting for the central love plot. In fact, *Exit the Actress* is the most “romantic” in a series of recent Gwyn novels.^5^ Without bending biographical facts too much, Parmar even manages to approximate the romance novel’s required Happily Ever After ending – its promise of a lasting, monogamous love relationship – by cutting her narrative off at the precise point when, historically, King Charles’s earlier mistress Barbara Palmer falls out of favour and shortly before Gwyn’s great rival (or co-mistress) Louise de Keroualle is about to enter the scene.

However, with its bustling urban setting, its contemporary diction, its quirky humour, and its heroine’s serial dating efforts, *Exit the Actress* is, in fact, more reminiscent of another popular genre that Stephanie Harzewski has termed “Postmodernism’s Last Romance” (24): the chick lit novel.

This morning (after lengthy debate) I wore my new white muslin gown (Rochester’s choice) with the cream *pointe*, blue sash, and my delicate little silver mules – lovely! (Teddy’s choice). But nothing! No response. The king does not seem to notice me at all. Grumble. (Parmar 287)

Its episodic form and informal first-person diary voice in fact associate *Exit the Actress* closely with Helen Fielding’s *Bridget Jones’s Diary* – one of the *ur*-texts of postfeminist chick lit:

Ugh. Cannot face thought of going to work. Only thing which makes it tolerable is thought of seeing Daniel again […] Love his wicked dissolute air, while being v. successful and clever […] Think might wear short black skirt tomorrow. (Fielding 17–19)

Nightmare day in office. Watched the door for Daniel all morning: nothing. (28)

The resemblance between the two novels is reinforced by the trope of the gay best friend (in *Exit the Actress* it is Edward “Teddy” Kynaston) and a meddling, judgmental mother figure, here in the guise of Queen Henrietta Maria, whose letters to and about the king provide much of the novel’s humour: “*For heaven’s sake, Charles, stop frolicking through the countryside like a milkmaid and get a tighter rein on your government*” (Parmar 250). On Queen Catherine’s infertility, for instance, she writes: “*We certainly know the problem does not lie with Charles – at least he has shown himself capable of this much*” (92).

Similarly, King Charles’s letters to his sister Minette in *Exit the Actress* serve to reveal aspects of the king’s private life, which is again done primarily in a light-hearted, humorous tone:

*Yes, I have attempted conjugal duties – no, it did not go well, although I do hope to entertain her better than the Monsieur did you. Your nightmarish account of that encounter haunts me still; I cannot imagine being unattracted to women – one or two perhaps, but* all*? Mystifying.* (33)

Parmar’s Nell also repeatedly goes on shopping sprees with her girl-friends (280, 316, 345), thus playing to another trope of postfeminist popular culture: that of the female consumer, which, according to Yvonne Tasker and Diane Negra, envisions “consumption itself as both therapeutic and transformative” (10). Parmar’s novel fits Munford and Waters’ description of postfeminist TV shows set in the past as typically marked by the “glorification of old-style femininities and masculinities” (28) and tending to “present affluent worlds in which women have limited power, but fabulous clothes” (ibid.).

Despite the heroine’s preoccupation with finding Mr Right, *Exit the Actress* is, like other postfeminist texts, repeatedly haunted by the “breaking through of a feminist consciousness” (Whelehan xi), which it projects onto its historical protagonist. Star actor Charles Hart, Nell’s first “date,” initially forbids Nell to exercise her talent as a comedienne on the grounds that he “will not have her laughed at” (147) but finally relents, on the condition that he play opposite her. “I know I should not but I resent his intrusion,” Nell writes. “Can I not stand alone as an actress, unclaimed and unsupervised?” (148). Parmar also has Nell protest again and again how much her profession means to her: “They […] cheer for me as the curtain comes down. It is an intoxicating thing to feel their love. It keeps me strong. It keeps me safe. No man can take this from me” (215). When King Charles does “take it from her” at the end of the novel, asking her to leave the stage when she is expecting his child, she first resents his attempt at controlling her but then agrees after joyfully realizing that it is his great love and concern for her health that is making him anxious. In this way, Parmar presents her seventeenth-century protagonist as a distinctly modern career woman but ultimately retains the conservative notion, familiar from historical romance novels, of woman’s development as geared towards finding their ultimate fulfilment in a monogamous love relationship.

## conclusion

Like many other biographical novels about women artists published these days, Galloway’s and Parmar’s biofictions both constitute attempts at telling stories about women that resonate across the centuries. As has been demonstrated, their interpretations of what it meant to be a woman artist in a period long gone are shaped considerably by the novelists’ freedom to draw on biographical as well as fictional modes of writing, and to deliberately cross the lines between fact and fiction in their work.

Although *Clara* and *Exit the Actress* resemble each other in the way they experiment with typography and layout, they differ markedly as regards the biographical and fictional subgenres in which they participate and, hence, in their gender politics. This difference, I suggest, is symptomatic of a time when feminist narratives of progress co-exist with a postfeminist nostalgia for “a lost, uncomplicated past” (Whelehan 11) and the “glorification of old-style femininities” (Munford and Waters 28). *Clara* focuses on its heroine’s struggle for her musical career in the spirit of feminist biography and points to the inaccessibility of female experience and hence the difficulty of writing a woman’s life, which also turns Galloway’s novel into an example of fictional metabiography. The fragmentation of Parmar’s novel, by contrast, serves mostly to add some historical colour to her narrative and break the first-person solipsism of the diary to introduce other voices, chiefly for the purpose of humour. While *Exit the Actress* is occasionally haunted by feminist ideas, it can be understood more readily in terms of both the historical romance novel and its postfeminist offspring the chick lit novel. Together, the two novels analysed demonstrate that biographical fictions about historical women, although concerned ostensibly with past lives, can serve as a barometer of shifting gender discourses of the twenty-first century.

## notes

1 On the rise of biographical fiction in the twentieth century, see Middeke; Lackey.

2 Feminist critics’ preoccupation with “situated knowledge” can also be read in this light and is often discussed in the work of feminist biographers; cf., for example, Stanley 250.

3 For a more detailed analysis of the way in which Galloway projects contemporary feminist ideas onto historical characters and events, see Novak, “Father and Daughter Across Europe.”

4 In an interview, Janice Galloway points out that while her novel is based on six years of biographical research and thus contains a wealth of factual material, she occasionally took liberties with her sources and made up some of the lines in the letters the novel purports to cite in translation (Galloway Interview). The reader has no way of telling whether or not her lines are faithful to the source material (unless s/he compares them with an edition of the Schumanns’ letters). Notwithstanding the uncertain status of the letters in *Clara*, I suggest that the very fact that some passages in the novel are narrated in the form of letters points to the biographer's practice of piecing a life together from the available life-writing records and thus serves as a reminder that Galloway's novel narrates the life of a historical person and is based on biographical research.

5 For an analysis of Diane Haeger's *The Perfect Royal Mistress* (2007), Susan Holloway Scott's *The King's Favorite* (2008) and Gillian Bagwell's *The Darling Strumpet* (2011), see Novak, “Nell Gwyn in Contemporary Romance Novels.”
